# The power of Drosophila genetics in studying insect toxicology and chemical ecology

**DOI:** 10.1007/s44297-023-00012-x

**Published:** 2023-11-27

**Authors:** Jia Huang, Youngseok Lee

**Affiliations:** 1 Institute of Insect Sciences, Zhejiang University, Hangzhou, 310058 China; 2 Department of Bio & Fermentation Convergence Technology, Kookmin University, Seoul, 02707 Republic of Korea

**Keywords:** Insect toxicology, Chemical ecology, *Drosophila melanogaster*, Genetics, Insecticide, Receptor

## Abstract

Insect toxicology and chemical ecology are inherently interconnected disciplines, both dedicated to unraveling the intricate relationships between insects and the diverse array of chemical compounds that pervade their surroundings. *Drosophila melanogaster*, owing to its genetic and physiological similarities to other insects, serves as a robust model system in the study of insect toxicology. Moreover, state-of-the-art techniques in Drosophila neurobiology have extensively probed the chemosensory system of insects, providing significant insights into their adaptation to chemical environments. In this review, we emphasize the advancements achieved through the application of Drosophila genetics in investigations spanning both of these fields, significantly enhancing our understanding of the mode of action and resistance mechanisms of insecticides, as well as unraveling the molecular and cellular mechanisms underlying insect chemosensation and associated behaviors. The profound insights derived through this tiny fly not only enrich our understanding of the broader world of insects but also hold the potential to develop more effective and sustainable strategies for pest management.

## Introduction

*Drosophila melanogaster*, commonly known as the fruit fly, has served as a model organism in genetics research for well over a century. The utilization of Drosophila genetics has proven invaluable in exploring fundamental biological processes such as development, behavior, and disease, as well as more applied fields such as toxicology and chemical ecology. One of the key advantages of employing *D. melanogaster* as a model organism lies in its well-characterized genome and the availability of powerful genetic tools. The fruit fly possesses a relatively small genome that lends itself to facile manipulation through techniques such as gene editing and RNA interference. Additionally, *D. melanogaster* exhibits a short generation time, facilitating rapid genetic analysis and high-throughput screening. The application of Drosophila genetics has yielded numerous pivotal discoveries in biological research, encompassing the identification of key developmental genes, the elucidation of the role of genetic mutations in disease, and the unraveling of molecular mechanisms underlying behavior and memory [1].

Insect toxicology and chemical ecology are closely related fields that both focus on understanding the interactions between insects and the chemical compounds present in their environment, but they approach this interaction from slightly different angles. Insect toxicology primarily deals with the study of how chemical substances, including insecticides and other toxic compounds, affect insects. It aims to understand the mechanisms of toxicity, how insects develop resistance to toxic substances, and the impact of these compounds on insect populations, ecosystems, and even human health. Insect chemical ecology, on the other hand, focuses on the role of chemical compounds in mediating interactions between insects and their environment. It investigates how insects use chemical cues for finding mates, locating suitable habitats, identifying hosts or prey, and avoiding predators. Therefore, comprehending insect toxicology and chemical ecology is important for developing effective pest management strategies that minimize the use of harmful chemicals. Furthermore, understanding the ecological roles of insects and their interactions with other organisms is equally vital.

Here, we compile and organize existing research about the applications of Drosophila genetics in insect toxicology and chemical ecology, serving as a centralized resource for researchers, students, and professionals in these fields. This consolidation helps individuals access a comprehensive overview of the state of the art in this specific area of study and allows for the exchange of ideas and methodologies between researchers to encourage interdisciplinary collaboration.

### Elucidating the mode of action of insecticides with Drosophila genetics

Chemical pesticides have been widely employed for pest control in agriculture, horticulture, forestry, as well as residential and urban areas. They have also played a crucial role in preventing the transmission of vector-borne diseases that affect both humans and animals. While the modes of action of most insecticides are known (www.irac-online.org), the precise molecular targets still remain elusive. Merely establishing an in vitro biochemical interaction between an insecticide and a protein is insufficient to confirm that the protein is indeed the target responsible for the insecticidal effect in vivo. Genetic evidence, demonstrating the impact of mutating the candidate receptor, is essential before conclusively identifying a specific protein as the target of an insecticide. Therefore, the utilization of forward/reverse genetics in *D. melanogaster* has proven to be a powerful approach in identifying protein targets for insecticides (Table 1). In cases where an insecticide does not exhibit toxicity towards flies, behavioral assays can be employed to characterize potential targets. For instance, climbing assays have been used to identify a *Drosophila* TRPV channel as the target for two insecticides, pymetrozine and pyrifluquinazon [2]. Similar strategies have also revealed the molecular target of flonicamid to be nicotinamidase [3]. Behavioral assays involving *Drosophila* null mutants of octopamine receptors have pinpointed Octβ2R, a receptor subtype, as the sole target of amitraz *in vivo* [4].

**Table 1:** Insecticide molecular targets identified and/or confirmed with Drosophila genetics

IRAC groups	Molecular targets	Drosophila strains	Reference
**2** GABA-gated chloride channel blockers	Rdl	A301S point-mutation allele	[5, 6]
**4** Nicotinic acetylcholine receptor competitive modulators	α1α2β1β2; α1β1β2; α3β1; α1α3β1 heterooligomers	α1, α2, α3, β2 null and β1 R81T point-mutation alleles	[7]
**5** Nicotinic acetylcholine receptor allosteric modu1 lators—Site I	α6 homooligomer	*α6* null allele	[7, 8]
**6** Glutamate-gated chloride channel allosteric modulators	GluCl	P299S point-mutation allele	[9]
**7** Juvenile hormone mimics	Met	*Met* null alleles	[10, 11]
**9** Chordotonal organ TRPV channel modulators	Nan/Iav	*Nan* and *Iav* null alleles	[2]
**10** Mite growth inhibitors affecting CHS1; **15** Inhibitors of chitin biosynthesis affecting CHS1	CHS1	I1056M/F point-mutation alleles	[12]
**14** Nicotinic acetylcholine receptor channel blockers	α6 homooligomer	*α6* null allele	unpublished
**19** Octopamine receptor agonists	Octβ2R	*Octβ2R* null allele	[4]
**29** Chordotonal organ nicotinamidase inhibitors	Naam	A265E point-mutation allele	[3]

Actually, the mode of action of insecticides is well conserved between *D. melanogaster* and other insects, probably because insecticides disrupt essential physiological functions. For instance, the nAChR gene family, encoding the direct targets of neonicotinoids, spinosyns and many other insecticides, exhibits slow evolution, and the core groups of nAChR subunits exhibit significant conservation across diverse insect species, spanning approximately 300 million years of evolution, underscoring their essential functions in the nervous system. The majority of Drosophila nAChR subunit genes have one-to-one orthologs in the genomes of other insects, and the sequence identities between these orthologs are likewise considerable. For some subunit genes, even alternative splicing and RNA editing are conserved [7].

Besides utilizing various target gene alleles, Drosophila offers sophisticated genetic toolboxes that enable the manipulation of candidate target genes and target-expressing neurons with high spatial and temporal resolution. For example, using UAS-controlled transgenes that express RNAi-inducing or ORF constructs can lead to tissue-specific RNAi or overexpression. Another important tool in *D. melanogaster* is the thermogenetics reagents, such as *UAS-trpA1* and *UAS-Shibire*^ts^. Expressing the thermosensitive cation channel Drosophila TRPA1 with the Gal4/UAS system to acutely hyperstimulate neurons expressing Octβ2R within a narrow time frame mimics the effects of amitraz on target pests, providing evidence that in vivo pharmacological activation of Octβ2R by amitraz leads to toxicity and eventual mortality [4]. Electrophysiological studies conducted on native tissues or recombinant receptors have demonstrated that low concentrations of neonicotinoids can inhibit nAChR, while higher concentrations result in receptor activation. Consequently, it has remained unclear whether the insecticidal activity stems from nAChR inhibition or activation in vivo. However, through the utilization of *Drosophila* thermogenetics tools, it has been discovered that transient artificial activation, rather than inhibition, of nAChR-expressing neurons is sufficient to induce symptoms resembling neonicotinoid poisoning in flies. Hence, the overall effect of neonicotinoids involves neuronal depolarization through nAChR activation, which is more physiologically relevant [7].

### Drosophila genetics as a powerful tool for studying insecticide resistance mechanisms

Invertebrate pest control faces a significant global challenge due to the prevalence of insecticide resistance, with over 600 different insect and mite species demonstrating resistance to at least one insecticide. Moreover, there are documented cases of resistance to more than 335 insecticides/acaricides. To address the potential failure of insecticide-based control methods, it is imperative to understand the underlying resistance mechanisms, which typically include behavioral, penetration, metabolic, and target-site resistance. The majority of the research conducted in the field to date has utilized the genetic tools and resources available in *D. melanogaster*, although the advent of CRISPR/Cas9 genome editing now allows for gene modifications in pests. Introducing point mutations identified in target genes of resistant pest populations into homologous sites in Drosophila is quick and straightforward, enabling genetic confirmation of the causal relationships between genotypes and resistance phenotypes (Table 2). Additionally, numerous reports have indicated that insecticide resistance is associated with variations in the overexpression of metabolic enzymes such as cytochrome P450s, carboxylesterases, glutathione-S-transferases, and UDP-glucuronosyltransferases. However, establishing a definitive causal link between overexpression and resistance has often lacked supporting evidence. Therefore, the controlled overexpression of metabolic genes from pests into *Drosophila* has proven to be a valuable tool in establishing connections between enzyme activity and resistance (Table 3).

**Table 2:** Target-site resistance mutations experimentally confirmed with Drosophila genetics

Insecticides/Targets	Species	Resistance alleles	Resistance ratios in Drosophila mutants	Reference
Avermectins/GluCl	*Plutella xylostella*	V263I	27.1	[13]
	*Tetranychus urticae*	I321T	3	[14]
Diamides/RyR	*Chilo suppressalis*	Y4667C^a^	1.3–8.6	[15, 16]
		I4758M + Y4667C^a^	19.5–172.1	
		Y4667D^a^	6.2–117.2	
		I4758M + Y4667D^a^	21.2- 1542.8	
		I4758M^a^	3.3–22	
		Y4891F^a^	5.9–10.2	
	*Plutella xylostella;* *Tuta absoluta;* *Chilo suppressalis*	G4946E^b^	25.2–153.1	
	*Plutella xylostella;* *Tuta absoluta*	G4946V^b^	5.4–194.7	[17]
	*Plutella xylostella;* *Tuta absoluta;* *Chilo suppressalis; Spodoptera exigua; Spodoptera frugiperda*	I4790M^b^ = I4758M^a^	2.3–15.3	
Fipronil/Rdl	*Laodelphax striatellus; Sogatella furcifera*	A2’N	1099	unpublished
Benzoylureas/CHS1	*Plutella xylostella* *Culex pipiens*	I1042F/M^b^	111–15,625	[12, 18]
Etoxazole; Clofentezine; Hexythiazox/CHS1	*Tetranychus urticae*	I1042F^b^	1077	
Indoxacarb; Metaflumizone/Para	*Plutella xylostella;* *Tuta absoluta*	V1848I^c^	6–8.4	[19]
		F1845Y^c^	10.2–3441.2	
Pyrethroids; DDT/Para	*Aedes aegypti*	I1011M^c^	> 3	[20, 21]
		V1016G^c^	3	
	Many species	L1014F^c^	12.7	
Spinosyns/nAChR	*Frankliniella occidentalis; Thrips palmi; Tuta absoluta*	α6 G275E	62.2	[22]
Neonicotinoids/nAChR	*Myzus persicae; Aphis gossypii*	β1 R81T	23.9–398.3	[7, 23]
Spiromesifen; Spirodiclofen; Spirotetramat/Accase	*Bemisia tabaci*	A2083V	874–3616	[24]

^a^*Chilo suppressalis* numbering. ^b^*Plutella xylostella* numbering. ^c^Housefly numbering

**Table 3:** Metabolic resistance genes experimentally confirmed with Drosophila genetics

Species	Insecticides	Transgenes	GAL4 drivers	References
*Apis mellifera*	Chlorantraniliprole	*CYP9Q2/3*	Hsp70-GAL4	[25]
	Flupyradifurone	*CYP9Q2/3*		[26]
	Thiacloprid	*CYP9Q1/2/3*	Malp-tub-GAL4	[27]
*Nilaparvata lugens*	*Imidacloprid*	*CYP6ER1 variants*	Act5C-GAL4	[28]
		*CYP6ER1*		[29]
		*Mdr49-like*	Actin-GAL4	[30]
	Buprofezin	*CYP6ER1vA; CYP439A1*	da-GAL4	[31]
	Chlorpyrifos	*CarE17*	Tub-GAL80^ts^ + Tub-GAL4	[32]
	Pymetrozine	*CYP6CS1*	Actin-GAL4	[33]
*Myzus persicae*	Nicotine; clothianidin	*CYP6CY3*	Direct insertion	[34]
	Sulfoxaflor	*CYP380C40; UGT344P2*	Act5C-GAL4	[35]
*Bemisia tabaci*	Cyantraniliprole	*CYP6CX3*	Actin-GAL4	[36]
	Imidacloprid	*CYP402C1*		[37]
	Imidacloprid; Nitenpyram	*CYP6CM1*	HR-GAL4	[38]
*Plutella xylostella*	chlorantraniliprole	*FMO2*	Act5C-GAL4	[39]
	β-cypermethrin; phoxim bifenthrin; chlorpyrifos; fenvalerate; malathion;	*αE14*	Actin-GAL4	[40]
	β-cypermethrin; phoxim	*αE8*		[41]
	β-cypermethrin; chlorantraniliprole	*CYP6BG1*		[42]
*Anopheles coluzzii*	Permethrin; DDT	*GSTe2*	Act5C-GAL4	[43]
*Anopheles gambiae*	Deltamethrin; DTT permethrin; bendiocarb	*Cyp6M2; CYP6P3*	Act5C-GAL4	[44]
	DDT	*GstE2*	HR-GAL4	[38]
*Anopheles funestus*	Deltamethrin	*CYP9J11*	Act5C-GAL4	[45]
	Deltamethrin; permethrin	*CYP6P9a; CYP6P9b*		[46, 47]
		*CYP6M7*		[48]
	Bendiocarb	*CYP6P9a; CYP6P9b*		[49]
*Aedes albopictus*	Haedoxan A	*CYP304A1*	Tub-Gal80^ts^ + Tub-Gal4	[50]
	Deltamethrin; etofenprox	*CYP6P12*	Act5C-GAL4	[51]
*Aedes aegypti*	Deltamethrin	*CYP9J28; CYP6BQ23*	HR-GAL4	[20]
	Permethrin	*CYP4D24*	Act5C-GAL4	[52]
*Anopheles albimanus*	α-cypermethrin; Deltamethrin	*CYP6P5*	Act5C-GAL4	[53]
*Musca domestica*	Permethrin	*CYP4S24; CYP6A36; CYP6D10*	Act5C-GAL4	[54]
	propoxur	*CYP6G4*	HR-GAL4; Act-GAL4	[55]
*Aphis gossypii*	Thiamethoxam	*CYPC6Y9; CYP4CK1; CYP6DB1; CYP6CZ1*	Act5C-GAL4	[56]
	Imidacloprid	*CYPC6Y9; CYP6CY22; CYP6CY18; CYP6D*		
	cyantraniliprole	*CYP380C6; CYP4CJ1*		[57]
	Spirotetramat	*CYP380C6; CYP4CJ1; CYP6DA2; CYP6CY7; CYP6CY21*	Esg-GAL4	[58]
*Spodoptera exigua*	chlorpyrifos	*CYP321A16; CYP332A1*	Act5C-GAL4	[59]
*Spodoptera litura*	Indoxacarb	*COE090; COE050; COE093; COE074*	Tub-GAL4	[60]
*Spodoptera frugiperda*	tricin	*CYP321A9*	Act5C-GAL4	[61]
*Ceratitis capitata*	Deltamethrin; λ-cyhalothrin	*CYP6A51*	HR-GAL4	[62]
*Bombus terrestris*	Thiacloprid	*CYP9Q4/5*	Hsp70-Gal4	[27]
	Thiacloprid; acetamiprid	*CYP9Q6*		[63]
*Osmia bicornis*	Thiacloprid	*CYP9BU1; CYP9BU2*	Hsp70-Gal4	[64]
*Lucilia cuprina*	Diazinon; malathion	*αE7*	HR-GAL4	[38]
*Tribolium castaneum*	Deltamethrin	*CYP6BQ9*	CNS-GAL4; Act5C-GAL4	[65]
*Bactrocera dorsalis*	malathion	*GSTe8-B*	da-Gal4	[66]
*Tetranychus urticae*	Fenpyroximate	*CYP392A11*	HR-GAL4	[67]
	Abamectin	*CYP392A16*		[68]

### Drosophila genetics as a model system for studying the chemical ecology of insects

Drosophila genetics has also been employed to investigate the field of chemical ecology in insects. Chemical ecology focuses on studying the interactions between organisms and their chemical environment, including the roles of chemicals in communication, defense, and other ecological interactions. Insect taste and odor receptors are very sensitive detectors to find nutritious food, mates, and safe oviposition sites or avoid any potential predators. *D. melanogaster* has been used as a model organism in a variety of chemical ecology studies, including those related to pheromones, food odorants/tastants, and plant volatiles/non-volatiles. Following the first identification of the insect taste or odor receptors in *D. melanogaster*, similar receptors have been identified in many other insects, including the silk moth, *Bombyx mori*, the malaria vector mosquito *Anopheles gambiae*, and the honey bee *Apis mellifera*.

One advantage of using Drosophila genetics in chemical ecology studies is the ability to identify specific genes and pathways involved in chemical sensing and response. For example, genetic screens have been used to identify chemoreceptors and other genes involved in the detection of specific chemical cues. Additionally, Drosophila genetics allows for the manipulation of specific genes or pathways to investigate their roles in chemical communication and other behaviors.

Insects commonly employ semiochemicals to communicate within their own species or with other species. These semiochemicals include pheromones, allomones, and kairomones. Food trail pheromones, alarm pheromones, and sex pheromones are examples that can significantly influence behavior and physiology. The production of allomones and kairomones allows insects to avoid harmful food sources or predators. Drosophila genetics has been instrumental in identifying the receptor of 11-*cis*-Vaccenyl Acetate (cVA) as a volatile sex pheromone. Furthermore, there are many contact-mediated pheromones, such as the male dominant monoalkenes, (*Z*)-7-tricosene and (*Z*)-9-tricosene, and the female specific (7*Z*,11*Z*)-heptacosadiene. These pheromones can be studied as aggregation pheromones to gain insights into their chemical communication. Research involving Drosophila genetics and various tools in chemical ecology provides not only an understanding of how to respond to specific chemicals but also insight into how the chemical signals integrate into the higher brain center.

Furthermore, the use of Drosophila genetics and many research tools in chemical ecology studies allows for comparisons across species. By studying the genetics and behavior of Drosophila in response to specific chemicals, researchers can gain insights into the evolution of chemical communication and other ecological interactions across different insect species.

### Identification of gustatory receptor for various tastants in Drosophila

Taste organs are broadly distributed, such as the mouth parts labellum, legs, wing margins, and a female ovipositor as external organs. In addition, the pharynx also houses gustatory receptor neurons (GRNs) as internal organs. *D. melanogaster* has 31 taste sensilla in each hemisphere. A taste sensillum has a pore to have both chemosensory and mechanosensory cells. The sensilla on the labellum are the most well studied taste sensilla, categorizing the bristles and the taste pegs. Each taste bristle is typically innervated by two or four bipolar chemosensory neurons and a mechanosensory neuron. The taste sensilla can be categorized as long (L), intermediate (I), and short (S)-types, depending on the size of the bristles. Each bristle was analyzed by the tip recording technique, making contact with the pore at the tip of the sensillum with the taste stimulus and an electrolyte. Experiments with various tastants distinguished at least four types of GRNs such as sweet-sensing, water-sensing, bitter-sensing, and salt-sensing GRNs. Alkaline-sensing GRNs have recently been identified. This finding suggests that each type of bristle may be more diverse and complex than previously thought, leaving the possibility of discovering uncharacterized GRNs in the future.

During the last two decades, many research groups have deorphanized GRs (Table 4). For example, GR43a has been identified as a fructose receptor that functions in the brain to detect fructose levels in hemolymph [69]. The Drosophila genome contains nine sweet GRs, primarily responsible for detecting sugars and other attractive chemicals. GR8a, GR66a, and GR98b were first characterized as a full repertoire of L-canavanine receptors [70, 71].

**Table 4:** Information of the gustatory receptors required for detecting tastants

Taste	Stimulus	Receptors	Organs responsible for sensation	References
Sweet	Sucrose	*Gr64a,* Gr*64b, Gr64c, Gr64d, Gr64e,* and *Gr64f*	Labellum	[72–75]
	Maltose	*Gr64a,* Gr*64b, Gr64c, Gr64d,* *Gr64e,* and *Gr64f*	Labellum	[72–75]
	Glucose	*Gr5a, Gr61a, Gr64a, Gr64b, Gr64d, Gr64e,* and* Gr64f*	Labellum	[72–75]
	Fructose	*Gr64a, Gr64b, Gr64d, Gr64e,* and* Gr64f*	Labellum	[74, 75]
Bitter (Synthetic compounds)	Denatonium	*Gr22e, Gr32a, Gr33a, Gr59c,* and* Gr66a*	Labellum	[76, 77]
	DEET	*Gr32a, Gr33a, Gr66a,* and *Gr89a*	Labellum	[78, 79]
	IR3535	*Gr47a*	Labellum	[80]
Bitter (Natural or plant derived compounds)	Caffeine	*Gr33a, Gr39a.a, Gr66a,* and *Gr93a*	Labellum	[77, 81, 82]
	L-canavanine	*Gr8a*, *Gr66a,* and* Gr98b*	Labellum	[70, 71]
	Strychnine	*Gr22e, Gr32a, Gr33a, Gr47a,* and* Gr66a*	Labellum	[77, 83, 84]
	Saponin	*Gr28b.c*	Labellum	[85]
	Nicotine	*Gr10a, Gr32a,* and* Gr33a*	Labellum	[86]
	Cucurbitacin B	*Gr33a*	Labellum	[87]
	Azadirachtin	*Gr32a* and* Gr33a*	Labellum	[77]
	Umbelliferon	*Gr33a, Gr39a, Gr66a,* and* Gr93a*	Labellum	[77, 88]
	Quinine	*Gr32a, Gr33a,* and* Gr66a*	Labellum	[77]
	Histamine	*Ir76b, Gr9a, Gr22e,* and *Gr98a*	Labellum	[89, 90]
Salty	High salt (Aversive)	*Ir7c, Ir25a,* and *Ir76b*	Labellum	[91]
	Low salt (Attractive)	*Ir25a, Ir56b,* and* Ir76b*	Labellum, leg	[92, 93]
Sour	Acetic acid (Aversive)	*Ir7a*	Labellum	[94]
	Carboxylic acid (lactic acid, citric acid, glycolic acid, and HCl) (Attractive)	*Gr5a, Gr61a, Gr64a-f, Ir25a,* and* Ir76b*	Labellum, leg	[95–97]
	HCl	*otopla*	labellum	[98]
Alkali	Hydroxide (Aversive)	*Alka*	Labellum, leg	[99]
Amino acid	Low concentration of arginine, lysine, proline (Aversive)	*Ir25a, Ir51b,* and* Ir76b*	Labellum	[100]
	Low and high concentration of valine, tryptophan, isoleucine, and leucine (Aversive)	*Ir25a, Ir51b,* and* Ir76b*	Labellum	[100]
	Low concentration of arginine, proline, lysine, low and high concentration of glycine, alanine, serine, threonine, and cysteine (Attractive)	*Gr5a, Gr61a, Gr64f, Ir20a, Ir25a,* and* Ir76b*	Labellum, leg	[100, 101]
	Low and high concentration of methionine and glutamine (Attractive)	*Ir25a* and* Ir76b*	Labellum	[100]
Metals	Copper and zinc (Aversive)	*Gr33a, Gr66a, Ir25a, Ir56b,* and* Ir76b*	Labellum	[102, 103]
Minerals	Calcium (Aversive)	*Ir25a, Ir62a,* and* Ir76b*	Labellum	[104]
Ammonia and polyamine	Ammonium salt, urea, and putrescine (Aversive)	*Ir25a, Ir51b,* and* Ir76b*	Labellum	[105]
	Polyamines (putrescine and cadaverine) (Aversive)	*Ir76b*	Labellum	[106]
Fatty acid	Hexanoic acid (Aversive)	*Gr32a, Gr33a,* and* Gr66a*	Labellum	[107]
	Hexanoic acid (Attractive)	*Ir56d*	Labellum	[107–109]
	Carbonation and fatty acids	*Ir25a, Ir56d, and Ir76b*	Labellum	[110]
	Hexanoic acid, octanoic acid, oleic acid, linoleic acid	*Gr64e, Gr64a-f, Ir25a,* and* Ir76b*	Labellum, leg	[111, 112]
Other attractive organic compounds	Glycerol	*Gr43a, Gr64a, Gr64b, Gr64c, Gr64d, Gr64e,* and* Gr64f*	Labellum	[74, 75, 111]
	Vitamin C	*Gr5a, Gr61a, Gr64b, Gr64c, Gr64e, Ir25a,* and* Ir76b*	Labellum	[74]

In insects, ionotropic receptors (IRs) are also very popular taste receptors that mainly function to detect salty and sour tastants (Table 4). Recent behavioral and physiological studies have revealed that GRs and IRs may function together to detect the same chemicals, such as amino acids, metal ions, hexanoic acids, and attractive carboxylic acids, although the pathway and the exact mechanism are not clear. One study utilized in vivo calcium imaging from the subesophageal zone (SEZ), which is the first place to receive all the peripheral taste information, to demonstrate the simultaneous activation and deactivation of IR25a and sweet GRs, respectively, in response to lactic acid stimuli [95]. Mutants lacking specific receptors exhibited defects in calcium imaging during the corresponding phases.

Most chemoreceptors, such as sweet and bitter taste receptors, detect a chemical in a dose-dependent manner. In contrast, depending on the concentration of salt and sour, *D. melanogaster* likes low concentrations and dislikes high concentrations. This preference is mediated by the specific GRNs that harbor the corresponding receptors. For example, IR56b and IR7c work in attractive or aversive GRNs to detect salt, respectively [91, 92]. Recent studies also provide the evidence that arginine, proline, and lysine among amino acids as well as low fatty acids such as hexanoic acid also work as attractive or aversive tastants depending on the concentrations.

Except GRs and IRs, other highly well conserved ion channels in the animal kingdom, such as pickpocket ion channels (PPKs), transient receptor potential ion channels (TRPs), otopetrins, and alkaliphile participate in contact chemosensation to detect water, pungent chemicals, inorganic protons, and basic solutions (Table 4).

### Identification of olfactory receptors for various odorants with Drosophila genetics

Olfactory receptor neurons (ORNs) in insects are found in the antennae and maxillary palps. Each sensillum contains ORN dendrites that can detect odors through pores. The axons of the ORNs innervate the glomeruli in the antennal lobes of the brain. The ORNs expressing the same receptor project to a single glomerulus in each hemisphere. They synapse with the projection neurons to transmit signals to the higher olfactory centers, such as the mushroom body and the lateral horn. The olfactory sensilla of the antennae can be divided into three morphological types: basiconic, coeloconic, and trichoid.

A bioinformatic search for *olfactory receptor* (*Or*) genes identified 60 *Or* genes that mainly function in the antennae or maxillary palps. *Orco* is unusually expressed in most olfactory neurons and is the most well conserved chemoreceptor gene in insects. ORCO is a coreceptor to detect specific odors with another specific OR, which results in the role of ORCO in the transport or function of another specific OR. Insect ORNs have been analyzed by extracellular recording techniques. Loss of *Or* genes does not affect the survival of ORNs. The deletion of *Or22a* and *Or22b* results in an empty neuron that is unresponsive to odors. Therefore, the empty neuron system has been widely used to identify unknown receptors by misexpressing them. ORs are required for detecting aversive odorants such as DEET, IR3535, picaridin, and pyrethrum as well as nutrient yeast, alcohol, and volatile sex pheromone, cVA (Table 5). IRs are another important clade to work in sensory neurons in ORNs but do not generally coexpress ORs. Olfactory sensory neurons housed in coeloconic sensilla do not express Orco and are tuned to acids, ammonia, and humidity. The most broadly expressed IRs (IR8a and IR25a) in the antennae mainly function to detect acids and organic compounds such as 1,4-diaminobutane, pyrrolidine, phenethylamine, ammonia, and polyamines (Table 5).

**Table 5:** Information about the olfactory receptors required for sensing odorants

Smell	Stimulus	Receptors	Organs responsible for sensation	References
Aversive	DEET	*Or59b* and *Orco* (*Or83b*)	Antennae	[113]
	DEET, IR3535, and picaridin	*Or42a*	Antennae and maxillary palp	[114]
	Geosmin	*Or56a*	Antennae	[115]
	Pyrethrum	*Or7a, Or42b, Or59b*, and *Or98a*	Antennae	[116]
Acidic	Carboxylic acid (acetic acid, propionic acid, and HCl)	*Ir8a* and *Ir64a*	Antennae	[117–119]
	2-oxovaleric acid, propionic acid, phenylacetic acid and phenylacetaldehyde	*Ir8a*	Antennae	[119, 120]
	Acetic acid, propionic acid, and butyric acid	*Ir75a*	Antennae	[121]
	Hydroxycinnamic acid	*Or71a*	Maxillary palp	[122]
Organic compounds	1,4-diaminobutane, pyrrolidine, phenethylamine, and ammonia	*Ir25a and Ir76b*	Antennae	[120]
Nutrient source	Yeast	*Or35a* and *Orco*	Antennae	[123]
*Drosophila* stress odorant (dSO)	CO_2_	*Gr21a* and* Gr63a*	Antennae	[124–126]
Alcohol	Hexanol	*Orco and Or35a*	Antennae	[120]
Courtship pheromones	Phenylacetic acid and phenyladehyde	*Ir84a*	Antennae	[127]
	9-tricosene	*Or7a*	Antennae	[128]
Ammonia and polyamines	Ammonium and amine	*Ir92a*	Antennae	[129]
	Polyamines (spermidine, putrescine, cadaverine, and others)	*Ir41a* and *Ir76b*	Antennae	[106]
Volatile Fatty acid (FA) pheromone	*cis*-vaccenyl acetate, cVA	*Or65a* and* Or67d*	Antennae	[130]
Olfactory responses of other insect species to plant derived compounds and pheromone components	*D. sechellia* to odor bouquet of noni fruit	*DsecOrco, DsecOr22a, DsecIr8a,* and *DsecIr75b*	Antennae	[131]
	*Locusta migratoria* to body pheromone phenylacetonitrile (PAN)	*LmOr70a*	Antennae	[132]
	*Scaptomyza flava* to Isothiocyanate (ITC) derived from mustard oil	*SflaOr67b1, SflaOr67b2,* and *SflaOr67b3*	Antennae	[133]
	*Campoletis chlorideae* to sex pheromone (tetradecanal (14:Ald) and 2-heptadecanone (2-Hep))	*CchlOR18 and CchlOR47*	Antennae	[134]

Recent interesting findings include a geosmin receptor, OR56a. Geosmin is an earthy or musty flavor from toxic microbes, triggering an aversive response in Drosophila flies. The geosmin detection system allows flies to generally inhibit feeding and oviposition [115]. In contrast, *D*. *sechillia* is an extreme specialist on *Morinda citrifolia* (noni fruit), while *D. melanogaster* is a generalist. The characterization of the Or22a pathway and comparative studies of the circuit from specialists and generalists provide how animal behavior evolves [131]. *DsecOrco*, *DsecOr22a*, *DsecIr8a*, and *DsecIr75b* are needed for detecting odor bouquets from noni fruit. *Scaptomyza flava*, an herbivorous leaf mining fly species in the family Drosophilidae, specializes in isothiocyanate (ITC)-producing plants, Brassicales. *Sfla* Or67bs mediate ITC responses [133], although *D. melanogaster* is known to detect ITC via TRPA1.

Recent pheromone studies in olfaction from parasitoids and locusts have provided interesting insights. *Campoletis chlorideae* is one of most common hymenopteran parasites emerging from *Helicoverpa armigera*. A recent study showed that *CchlOr18* and *CchlOr47* are selectively tuned to two female-derived pheromones, tetradecanal and 2-heptadecanone, to elicit strong responses from males [134]. These pheromones can be developed to control specific pests. In addition, cannibalism in migratory locusts is known to be mediated by phenylacetonitrile and its receptor, *LmOr70a* [132]. Researchers can gain insight into the mechanisms of chemical communication and other ecological interactions across diverse insect species.

## Perspectives

Our review emphasizes the pivotal role this model organism has played in advancing our understanding of insect responses to chemicals, including breakthroughs in the mode of action of insecticides, resistance mechanisms, and the molecular basis of chemosensation. Understanding how Drosophila research informs strategies for pest management, crop protection and sustainable agriculture is vital for addressing the practical challenges associated with chemical control of insect pests. This type of review can also help students and early-career scientists gain a deeper understanding of the foundational principles and recent advances. While genome modification becomes increasingly accessible in non-model species and related resources continue to accumulate, the value of *D. melanogaster* as a model organism for studying insect toxicology and chemical ecology is still expected to persist well into the future. The expanding repertoire of genetic and genomic resources, along with the accompanying technologies, presents numerous opportunities for researchers in this field.

## Data Availability

All data analyzed during this study are included within the paper.

## References

[1] Bellen HJ, Tong C, Tsuda H. 100 years of Drosophila research and its impact on vertebrate neuroscience: a history lesson for the future. Nat Rev Neurosci. 2010;11:514–22. 20383202 10.1038/nrn2839 PMC4022039

[2] Nesterov A, et al. TRP channels in insect stretch receptors as insecticide targets. Neuron. 2015;86:665–71. 25950634 10.1016/j.neuron.2015.04.001

[3] Qiao X, et al. An insecticide target in mechanoreceptor neurons. Sci Adv. 2022;8:eabq3132. 36417522 10.1126/sciadv.abq3132 PMC9683716

[4] Guo L, Fan XY, Qiao X, Montell C, Huang J. An octopamine receptor confers selective toxicity of amitraz on honeybees and Varroa mites. Elife. 2010;10:e68268. 10.7554/eLife.68268 PMC8313232 34263722

[5] Ffrench-Constant RH, Mortlock DP, Shaffer CD, MacIntyre RJ, Roush RT. Molecular cloning and transformation of cyclodiene resistance in Drosophila: an invertebrate g-aminobutyric acid subtype A receptor locus. Proc Natl Acad Sci USA. 1991;88:7209–13. 1651498 10.1073/pnas.88.16.7209 PMC52263

[6] Ffrench-Constant RH, Rocheleau TA, Steichen JC, Chalmers AE. A point mutation in a Drosophila GABA receptor confers insecticide resistance. Nature. 1993;363:449–51. 8389005 10.1038/363449a0

[7] Lu W, Liu Z, Fan X, Zhang X, Qiao X, Huang J. Nicotinic acetylcholine receptor modulator insecticides act on diverse receptor subtypes with distinct subunit compositions. PLoS Genet. 2022;18:e1009920. 35045067 10.1371/journal.pgen.1009920 PMC8803171

[8] Watson GB, et al. A spinosyn-sensitive Drosophila melanogaster nicotinic acetylcholine receptor identified through chemically induced target site resistance, resistance gene identification, and heterologous expression. Insect Biochem Mol Biol. 2010;40:376–84. 19944756 10.1016/j.ibmb.2009.11.004

[9] Kane NS, et al. Drug-resistant Drosophila indicate glutamate-gated chloride channels are targets for the antiparasitics nodulisporic acid and ivermectin. Proc Natl Acad Sci U S A. 2000;97:13949–54. 11095718 10.1073/pnas.240464697 PMC17681

[10] Wilson TG, Fabian J. A Drosophila melanogaster mutant resistant to a chemical analog of juvenile hormone. Dev Biol. 1986;118:190–201. 3095161 10.1016/0012-1606(86)90087-4

[11] Wilson TG, Ashok M. Insecticide resistance resulting from an absence of target-site gene product. P Natl Acad Sci USA. 1998;95:14040–4. 10.1073/pnas.95.24.14040 PMC24322 9826649

[12] Douris V, et al. Resistance mutation conserved between insects and mites unravels the benzoylurea insecticide mode of action on chitin biosynthesis. Proc Natl Acad Sci U S A. 2016;113:14692–7. 27930336 10.1073/pnas.1618258113 PMC5187681

[13] Sun X, et al. A novel V263I mutation in the glutamate-gated chloride channel of Plutella xylostella (L.) confers a high level of resistance to abamectin. Int J Biol Macromol. 2023;230:123389. 36706876 10.1016/j.ijbiomac.2023.123389

[14] Xue W, et al. Untangling a Gordian knot: the role of a GluCl3 I321T mutation in abamectin resistance in Tetranychus urticae. Pest Manag Sci. 2021;77:1581–93. 33283957 10.1002/ps.6215

[15] Huang JM, et al. Double ryanodine receptor mutations confer higher diamide resistance in rice stem borer, Chilo suppressalis. Pest Manag Sci. 2021;77:4971–9. 34223694 10.1002/ps.6539

[16] Huang 黄镜梅 JM, et al. Multiple target-site mutations occurring in lepidopterans confer resistance to diamide insecticides. Insect Biochem Mol Biol. 2020;121:103367. 32243905 10.1016/j.ibmb.2020.103367

[17] Douris V, et al. Investigation of the contribution of RyR target-site mutations in diamide resistance by CRISPR/Cas9 genome modification in Drosophila. Insect Biochem Mol Biol. 2017;87:127–35. 28669775 10.1016/j.ibmb.2017.06.013

[18] Grigoraki L, et al. Striking diflubenzuron resistance in Culex pipiens, the prime vector of West Nile virus. Sci Rep. 2017;7:11699. 28916816 10.1038/s41598-017-12103-1 PMC5601912

[19] Samantsidis GR, O’Reilly AO, Douris V, Vontas J. Functional validation of target-site resistance mutations against sodium channel blocker insecticides (SCBIs) via molecular modeling and genome engineering in Drosophila. Insect Biochem Mol Biol. 2019;104:73–81. 30572019 10.1016/j.ibmb.2018.12.008

[20] Samantsidis GR, et al. “What I cannot create, I do not understand”: functionally validated synergism of metabolic and target site insecticide resistance. Proc Biol Sci. 2020;287:20200838. 32453986 10.1098/rspb.2020.0838 PMC7287358

[21] Kaduskar B, et al. Reversing insecticide resistance with allelic-drive in Drosophila melanogaster. Nat Commun. 2022;13:291. 35022402 10.1038/s41467-021-27654-1 PMC8755802

[22] Zimmer CT, et al. A CRISPR/Cas9 mediated point mutation in the alpha 6 subunit of the nicotinic acetylcholine receptor confers resistance to spinosad in Drosophila melanogaster. Insect Biochem Molec. 2016;73:62–9. 10.1016/j.ibmb.2016.04.007 PMC4876769 27117524

[23] Homem RA, et al. Evolutionary trade-offs of insecticide resistance - the fitness costs associated with target-site mutations in the nAChR of Drosophila melanogaster. Mol Ecol. 2020;29:2661–75. 32510730 10.1111/mec.15503 PMC7496652

[24] Lueke B, et al. Identification and functional characterization of a novel acetyl-CoA carboxylase mutation associated with ketoenol resistance in Bemisia tabaci. Pestic Biochem Physiol. 2020;166:104583. 32448413 10.1016/j.pestbp.2020.104583

[25] Haas J, Glaubitz J, Koenig U, Nauen R. A mechanism-based approach unveils metabolic routes potentially mediating chlorantraniliprole synergism in honey bees, Apis mellifera L., by azole fungicides. Pest Manag Sci. 2022;78:965–73. 34734657 10.1002/ps.6706 PMC9299185

[26] Haas J, et al. A toxicogenomics approach reveals characteristics supporting the honey bee (Apis mellifera L.) safety profile of the butenolide insecticide flupyradifurone. Ecotoxicol Environ Saf. 2021;217:112247. 33901780 10.1016/j.ecoenv.2021.112247

[27] Manjon C, et al. Unravelling the molecular determinants of bee sensitivity to neonicotinoid insecticides. Curr Biol. 2018;28:1137–43 e1135. 29576476 10.1016/j.cub.2018.02.045 PMC5887109

[28] Zimmer CT, et al. Neofunctionalization of Duplicated P450 Genes Drives the Evolution of Insecticide Resistance in the Brown Planthopper. Curr Biol. 2018;28:268–74 e265. 29337073 10.1016/j.cub.2017.11.060 PMC5788746

[29] Pang R, Chen M, Liang Z, Yue X, Ge H, Zhang W. Functional analysis of CYP6ER1, a P450 gene associated with imidacloprid resistance in Nilaparvata lugens. Sci Rep. 2016;6:34992. 27721443 10.1038/srep34992 PMC5056347

[30] Wang LX, et al. Overexpression of ATP-binding cassette transporter Mdr49-like confers resistance to imidacloprid in the field populations of brown planthopper, Nilaparvata lugens. Pest Manag Sci. 2022;78:579–90. 34596946 10.1002/ps.6666

[31] Zeng B, et al. The overexpression of cytochrome P450 genes confers buprofezin resistance in the brown planthopper, Nilaparvata lugens (Stal). Pest Manag Sci. 2023;79:125–33. 36098067 10.1002/ps.7181

[32] Lu K, Li Y, Xiao T, Sun Z. The metabolic resistance of Nilaparvata lugens to chlorpyrifos is mainly driven by the carboxylesterase CarE17. Ecotoxicol Environ Saf. 2022;241:113738. 35679727 10.1016/j.ecoenv.2022.113738

[33] Wang LX, Tao S, Zhang Y, Jia YL, Wu SF, Gao CF. Mechanism of metabolic resistance to pymetrozine in Nilaparvata lugens: over-expression of cytochrome P450 CYP6CS1 confers pymetrozine resistance. Pest Manag Sci. 2021;77:4128–37. 33913602 10.1002/ps.6438

[34] Bass C, et al. Gene amplification and microsatellite polymorphism underlie a recent insect host shift. Proc Natl Acad Sci U S A. 2013;110:19460–5. 24218582 10.1073/pnas.1314122110 PMC3845143

[35] Pym A, et al. Overexpression of UDP-glucuronosyltransferase and cytochrome P450 enzymes confers resistance to sulfoxaflor in field populations of the aphid. Myzus persicae Insect Biochem Mol Biol. 2022;143:103743. 35202811 10.1016/j.ibmb.2022.103743

[36] Zhang Z, et al. Cytochrome P450 gene, CYP6CX3, is involved in the resistance to cyantraniliprole in Bemisia tabaci. J Agric Food Chem. 2022;70:12398–407. 36154000 10.1021/acs.jafc.2c04699

[37] Guo L, et al. Expression profile of CYP402C1 and its role in resistance to imidacloprid in the whitefly, Bemisia tabaci. Insect Sci. 2023;30:146–60. 35603806 10.1111/1744-7917.13081

[38] Daborn PJ, et al. Using Drosophila melanogaster to validate metabolism-based insecticide resistance from insect pests. Insect Biochem Mol Biol. 2012;42:918–24. 23023059 10.1016/j.ibmb.2012.09.003

[39] Mallott M, et al. A flavin-dependent monooxgenase confers resistance to chlorantraniliprole in the diamondback moth, Plutella xylostella. Insect Biochem Mol Biol. 2019;115:103247. 31626952 10.1016/j.ibmb.2019.103247 PMC6880784

[40] Li R, et al. Overexpression of PxalphaE14 Contributing to Detoxification of Multiple Insecticides in Plutella xylostella (L.). J Agric Food Chem. 2022;70:5794–804. 35510781 10.1021/acs.jafc.2c01867

[41] Li R, Zhu B, Shan J, Li L, Liang P, Gao X. Functional analysis of a carboxylesterase gene involved in beta-cypermethrin and phoxim resistance in Plutella xylostella (L.). Pest Manag Sci. 2021;77:2097–105. 33342080 10.1002/ps.6238

[42] Li X, Li R, Zhu B, Gao X, Liang P. Overexpression of cytochrome P450 CYP6BG1 may contribute to chlorantraniliprole resistance in Plutella xylostella (L.). Pest Manag Sci. 2018;74:1386–93. 29194968 10.1002/ps.4816

[43] Ibrahim SS, et al. Molecular drivers of insecticide resistance in the Sahelo-Sudanian populations of a major malaria vector Anopheles coluzzii. BMC Biol. 2023;21:125. 37226196 10.1186/s12915-023-01610-5 PMC10210336

[44] Edi CV, et al. CYP6 P450 enzymes and ACE-1 duplication produce extreme and multiple insecticide resistance in the malaria mosquito Anopheles gambiae. PLoS Genet. 2014;10:e1004236. 24651294 10.1371/journal.pgen.1004236 PMC3961184

[45] Riveron JM, et al. Genome-wide transcription and functional analyses reveal heterogeneous molecular mechanisms driving pyrethroids resistance in the major malaria vector Anopheles funestus across Africa. G3 (Bethesda). 2017;7:1819–32. 28428243 10.1534/g3.117.040147 PMC5473761

[46] Ibrahim SS, et al. Allelic variation of cytochrome P450s drives resistance to bednet insecticides in a major malaria vector. PLoS Genet. 2015;11:e1005618. 26517127 10.1371/journal.pgen.1005618 PMC4627800

[47] Riveron JM, et al. Directionally selected cytochrome P450 alleles are driving the spread of pyrethroid resistance in the major malaria vector Anopheles funestus. Proc Natl Acad Sci U S A. 2013;110:252–7. 23248325 10.1073/pnas.1216705110 PMC3538203

[48] Riveron JM, et al. The highly polymorphic CYP6M7 cytochrome P450 gene partners with the directionally selected CYP6P9a and CYP6P9b genes to expand the pyrethroid resistance front in the malaria vector Anopheles funestus in Africa. BMC Genomics. 2014;15:817. 25261072 10.1186/1471-2164-15-817 PMC4192331

[49] Mugenzi LMJ, et al. The duplicated P450s CYP6P9a/b drive carbamates and pyrethroids cross-resistance in the major African malaria vector Anopheles funestus. PLoS Genet. 2023;19:e1010678. 36972302 10.1371/journal.pgen.1010678 PMC10089315

[50] Pei Y, Hao H, Zuo Y, Xue Y, Aioub AAA, Hu Z. Functional validation of CYP304A1 associated with haedoxan A detoxification in Aedes albopictus by RNAi and transgenic drosophila. Pest Manag Sci. 2023;79:447–53. 36175391 10.1002/ps.7213

[51] Ishak IH, et al. The cytochrome P450 gene CYP6P12 confers pyrethroid resistance in kdr-free Malaysian populations of the dengue vector Aedes albopictus. Sci Rep. 2016;6:24707. 27094778 10.1038/srep24707 PMC4837359

[52] Reid WR, et al. Transcriptional analysis of four family 4 P450s in a Puerto Rico strain of Aedes aegypti (Diptera: Culicidae) compared with an Orlando strain and their possible functional roles in permethrin resistance. J Med Entomol. 2014;51:605–15. 24897853 10.1603/me13228

[53] Kusimo MO, et al. Pyrethroid resistance in the New World malaria vector Anopheles albimanus is mediated by cytochrome P450 CYP6P5. Pesticide Biochem Physiol. 2022;183:105061. 10.1016/j.pestbp.2022.105061 PMC9125164 35430064

[54] Li M, Feng X, Reid WR, Tang F, Liu N. Multiple-P450 gene co-up-regulation in the development of permethrin resistance in the house fly, musca domestica. Int J Mol Sci. 2023;24:3170. 10.3390/ijms24043170 PMC9959456 36834582

[55] Zhu J, Feng J, Tian K, Li C, Li M, Qiu X. Functional characterization of CYP6G4 from the house fly in propoxur metabolism and resistance. Pestic Biochem Physiol. 2022;187:105186. 36127048 10.1016/j.pestbp.2022.105186

[56] Lv Y, et al. Functional validation of the roles of cytochrome P450s in tolerance to thiamethoxam and imidacloprid in a field population of Aphis gossypii. J Agric Food Chem. 2022;70:14339–51. 36165284 10.1021/acs.jafc.2c04867

[57] Zeng X, et al. Resistance risk assessment of the ryanoid anthranilic diamide insecticide cyantraniliprole in Aphis gossypii Glover. J Agric Food Chem. 2021;69:5849–57. 34014075 10.1021/acs.jafc.1c00922

[58] Peng T, et al. Functional investigation of lncRNAs and target cytochrome P450 genes related to spirotetramat resistance in Aphis gossypii Glover. Pest Manag Sci. 2022;78:1982–91. 35092151 10.1002/ps.6818

[59] Hu B, et al. Xenobiotic transcription factors CncC and maf regulate expression of CYP321A16 and CYP332A1 that mediate chlorpyrifos resistance in Spodoptera exigua. J Hazard Mater. 2020;398:122971. 10.1016/j.jhazmat.2020.122971 32512455

[60] Shi Y, Li W, Zhou Y, Liao X, Shi L. Contribution of multiple overexpressed carboxylesterase genes to indoxacarb resistance in Spodoptera litura. Pest Manag Sci. 2022;78:1903–14. 35066991 10.1002/ps.6808

[61] He L, et al. Cytochrome P450s genes CYP321A9 and CYP9A58 contribute to host plant adaptation in the fall armyworm Spodoptera frugiperda. Pest Manag Sci. 2023;79:1783–90. 36627818 10.1002/ps.7355

[62] Tsakireli D, Riga M, Kounadi S, Douris V, Vontas J. Functional characterization of CYP6A51, a cytochrome P450 associated with pyrethroid resistance in the Mediterranean fruit fly ceratitis capitata. Pestic Biochem Physiol. 2019;157:196–203. 31153469 10.1016/j.pestbp.2019.03.022

[63] Troczka BJ, et al. Identification and functional characterisation of a novel N-cyanoamidine neonicotinoid metabolising cytochrome P450, CYP9Q6, from the buff-tailed bumblebee Bombus terrestris. Insect Biochem Mol Biol. 2019;111:103171. 31136794 10.1016/j.ibmb.2019.05.006 PMC6675907

[64] Beadle K, et al. Genomic insights into neonicotinoid sensitivity in the solitary bee Osmia bicornis. Plos Genet. 2019;15:e1007903. 10.1371/journal.pgen.1007903 PMC6375640 30716069

[65] Zhu F, et al. A brain-specific cytochrome P450 responsible for the majority of deltamethrin resistance in the QTC279 strain of Tribolium castaneum. Proc Natl Acad Sci U S A. 2010;107:8557–62. 20410462 10.1073/pnas.1000059107 PMC2889294

[66] Lu XP, Xu L, Meng LW, Wang LL, Niu J, Wang JJ. Divergent molecular evolution in glutathione S-transferase conferring malathion resistance in the oriental fruit fly, Bactrocera dorsalis (Hendel). Chemosphere. 2020;242:125203. 31678848 10.1016/j.chemosphere.2019.125203

[67] Riga M, et al. Functional characterization of the Tetranychus urticae CYP392A11, a cytochrome P450 that hydroxylates the METI acaricides cyenopyrafen and fenpyroximate. Insect Biochem Molec. 2015;65:91–9. 10.1016/j.ibmb.2015.09.004 26363294

[68] Riga M, Ilias A, Vontas J, Douris V. Co-Expression of a homologous cytochrome P450 reductase is required for in vivo validation of the tetranychus urticae CYP392A16-based abamectin resistance in Drosophila. Insects. 2020;11:829. 10.3390/insects11120829 PMC7761253 33255521

[69] Miyamoto T, Slone J, Song X, Amrein H. A fructose receptor functions as a nutrient sensor in the Drosophila brain. Cell. 2012;151:1113–25. 23178127 10.1016/j.cell.2012.10.024 PMC3509419

[70] Lee Y, Kang MJ, Shim J, Cheong CU, Moon SJ, Montell C. Gustatory receptors required for avoiding the insecticide L-canavanine. J Neurosci. 2012;32:1429–35. 22279227 10.1523/JNEUROSCI.4630-11.2012 PMC3356580

[71] Shim J, et al. The full repertoire of Drosophila gustatory receptors for detecting an aversive compound. Nat Commun. 2015;6:1–8. 10.1038/ncomms9867 PMC4660205 26568264

[72] Jiao Y, Moon SJ, Montell C. A Drosophila gustatory receptor required for the responses to sucrose, glucose, and maltose identified by mRNA tagging. Proc Natl Acad Sci. 2007;104:14110–5. 17715294 10.1073/pnas.0702421104 PMC1955822

[73] Jiao Y, Moon SJ, Wang X, Ren Q, Montell C. Gr64f is required in combination with other gustatory receptors for sugar detection in Drosophila. Curr Biol. 2008;18:1797–801. 19026541 10.1016/j.cub.2008.10.009 PMC2676565

[74] Shrestha B, Aryal B, Lee Y. The taste of vitamin C in Drosophila. EMBO Rep. 2023;24:e56319. 10.15252/embr.202256319 PMC10240197 37114473

[75] Freeman EG, Wisotsky Z, Dahanukar A. Detection of sweet tastants by a conserved group of insect gustatory receptors. Proc Natl Acad Sci. 2014;111:1598–603. 24474785 10.1073/pnas.1311724111 PMC3910600

[76] Sung HY, et al. Heterogeneity in the Drosophila gustatory receptor complexes that detect aversive compounds. Nat Commun. 2017;8:1–10. 29133786 10.1038/s41467-017-01639-5 PMC5684318

[77] Dweck HK, Carlson JR. Molecular logic and evolution of bitter taste in Drosophila. Curr Biol. 2020;30:17–30 e13. 31839451 10.1016/j.cub.2019.11.005 PMC6946858

[78] Shrestha B, Lee Y. Mechanisms of DEET gustation in Drosophila. Insect Biochem Mol Biol. 2021;131:103550. 33549816 10.1016/j.ibmb.2021.103550

[79] Lee Y, Kim SH, Montell C. Avoiding DEET through insect gustatory receptors. Neuron. 2010;67:555–61. 20797533 10.1016/j.neuron.2010.07.006 PMC2929391

[80] Shrestha B, Nhuchhen Pradhan R, Nath DK, Lee Y. Cellular and molecular basis of IR3535 perception in Drosophila. Pest Manag Sci. 2022;78:793–802. 34708523 10.1002/ps.6693

[81] Moon SJ, Köttgen M, Jiao Y, Xu H, Montell C. A taste receptor required for the caffeine response in vivo. Curr Biol. 2006;16:1812–7. 16979558 10.1016/j.cub.2006.07.024

[82] Lee Y, Moon SJ, Montell C. Multiple gustatory receptors required for the caffeine response in Drosophila. Proc Natl Acad Sci. 2009;106:4495–500. 19246397 10.1073/pnas.0811744106 PMC2657413

[83] Poudel S, Kim Y, Gwak J-S, Jeong S, Lee Y. Gustatory receptor 22e is essential for sensing chloroquine and strychnine in Drosophila melanogaster. Insect Biochem Mol Biol. 2017;88:30–6. 28751111 10.1016/j.ibmb.2017.07.007

[84] Lee Y, Moon SJ, Wang Y, Montell C. A Drosophila gustatory receptor required for strychnine sensation. Chem Senses. 2015;40:525–33. 26187906 10.1093/chemse/bjv038 PMC4580539

[85] Sang J, Rimal S, Lee Y. Gustatory receptor 28b is necessary for avoiding saponin in Drosophila melanogaster. EMBO Rep. 2019;20:e47328. 30622216 10.15252/embr.201847328 PMC6362386

[86] Rimal S, Lee Y. Molecular sensor of nicotine in taste of Drosophila melanogaster. Insect Biochem Mol Biol. 2019;111:103178. 31226410 10.1016/j.ibmb.2019.103178

[87] Rimal S, Sang J, Dhakal S, Lee Y. Cucurbitacin B activates bitter-sensing gustatory receptor neurons via gustatory receptor 33a in Drosophila melanogaster. Mol Cells. 2020;43:530. 32451368 10.14348/molcells.2020.0019 PMC7332364

[88] Poudel S, Kim Y, Kim YT, Lee Y. Gustatory receptors required for sensing umbelliferone in Drosophila melanogaster. Insect Biochem Mol Biol. 2015;66:110–8. 26524963 10.1016/j.ibmb.2015.10.010

[89] Aryal B, Lee Y. Histamine avoidance through three gustatory receptors in Drosophila melanogaster. Insect Biochem Mol Biol. 2022;144:103760. 10.1016/j.ibmb.2022.103760 35346814

[90] Aryal B, Lee Y. Histamine gustatory aversion in Drosophila melanogaster. Insect Biochem Mol Biol. 2021;134:103586. 33992752 10.1016/j.ibmb.2021.103586

[91] McDowell SA, Stanley M, Gordon MD. A molecular mechanism for high salt taste in Drosophila. bioRxiv. 2022. 10.1016/j.cub.2022.06.012 35772408

[92] Dweck HK, Talross GJ, Luo Y, Ebrahim SA, Carlson JR. Ir56b is an atypical ionotropic receptor that underlies appetitive salt response in Drosophila. Curr Biol. 2022;32:1776–87 e1774. 35294865 10.1016/j.cub.2022.02.063 PMC9050924

[93] Zhang YV, Ni J, Montell C. The molecular basis for attractive salt-taste coding in Drosophila. Science. 2013;340:1334–8. 23766326 10.1126/science.1234133 PMC4091975

[94] Rimal S, Sang J, Poudel S, Thakur D, Montell C, Lee Y. Mechanism of acetic acid gustatory repulsion in Drosophila. Cell Rep. 2019;26:1432–42 e1434. 30726729 10.1016/j.celrep.2019.01.042 PMC6490183

[95] Stanley M, Ghosh B, Weiss ZF, Christiaanse J, Gordon MD. Mechanisms of lactic acid gustatory attraction in Drosophila. Curr Biol. 2021;31:3525–37 e3526. 34197729 10.1016/j.cub.2021.06.005

[96] Shrestha B, Lee Y. Mechanisms of carboxylic acid attraction in Drosophila melanogaster. Mol Cells. 2021;44:900. 34711686 10.14348/molcells.2021.0205 PMC8718364

[97] Chen Y, Amrein H. Ionotropic receptors mediate Drosophila oviposition preference through sour gustatory receptor neurons. Curr Biol. 2017;27:2741–50 e2744. 28889974 10.1016/j.cub.2017.08.003 PMC5680077

[98] Ganguly A, Chandel A, Turner H, Wang S, Liman ER, Montell C. Requirement for an otopetrin-like protein for acid taste in Drosophila. Proc Natl Acad Sci. 2021;118:e2110641118. 10.1073/pnas.2110641118 PMC8713817 34911758

[99] Mi T, et al. Alkaline taste sensation through the alkaliphile chloride channel in Drosophila. Nat Metab. 2023;5:466-80. 10.1038/s42255-023-00765-3 PMC10665042 36941450

[100] Aryal B, Dhakal S, Shrestha B, Lee Y. Molecular and neuronal mechanisms for amino acid taste perception in the Drosophila labellum. Curr Biol. 2022;32:1376–86. 10.1016/j.cub.2022.01.060 35176225

[101] Ganguly A, et al. A molecular and cellular context-dependent role for Ir76b in detection of amino acid taste. Cell Rep. 2017;18:737–50. 28099851 10.1016/j.celrep.2016.12.071 PMC5258133

[102] Xiao S, Baik LS, Shang X, Carlson JR. Meeting a threat of the Anthropocene: taste avoidance of metal ions by Drosophila. Proc Natl Acad Sci. 2022;119:e2204238119. 35700364 10.1073/pnas.2204238119 PMC9231609

[103] Luo R, et al. Molecular basis and homeostatic regulation of zinc taste. Protein Cell. 2022;13:462–9. 33891304 10.1007/s13238-021-00845-8 PMC9095774

[104] Lee Y, Poudel S, Kim Y, Thakur D, Montell C. Calcium taste avoidance in Drosophila. Neuron. 2018;97:67–74 e64. 29276056 10.1016/j.neuron.2017.11.038 PMC5777298

[105] Dhakal S, Sang J, Aryal B, Lee Y. Ionotropic receptors mediate nitrogenous waste avoidance in Drosophila melanogaster. Commun Biol. 2021;4:1–10. 34773080 10.1038/s42003-021-02799-3 PMC8589963

[106] Hussain A, et al. Ionotropic chemosensory receptors mediate the taste and smell of polyamines. PLoS Biol. 2016;14:e1002454. 27145030 10.1371/journal.pbio.1002454 PMC4856413

[107] Roshani Nhuchhen Pradhan BS, Youngseok Lee. Molecular basis of hexanoic acid taste in Drosophila melanogaster. Mol Cells. 2023;46:451–60. 10.14348/molcells.2023.0035 PMC10336273 37202372

[108] Brown EB, Shah KD, Palermo J, Dey M, Dahanukar A, Keene AC. Ir56d-dependent fatty acid responses in Drosophila uncover taste discrimination between different classes of fatty acids. Elife. 2021;10:e67878. 33949306 10.7554/eLife.67878 PMC8169106

[109] Tauber JM, Brown EB, Li Y, Yurgel ME, Masek P, Keene AC. A subset of sweet-sensing neurons identified by IR56d are necessary and sufficient for fatty acid taste. PLoS Genet. 2017;13:e1007059. 29121639 10.1371/journal.pgen.1007059 PMC5697886

[110] Sánchez-Alcañiz JA, et al. An expression atlas of variant ionotropic glutamate receptors identifies a molecular basis of carbonation sensing. Nat Commun. 2018;9:1–14. 30315166 10.1038/s41467-018-06453-1 PMC6185939

[111] Kim H, Kim H, Kwon JY, Seo JT, Shin DM, Moon SJ. Drosophila Gr64e mediates fatty acid sensing via the phospholipase C pathway. PLoS Genet. 2018;14:e1007229. 29420533 10.1371/journal.pgen.1007229 PMC5821400

[112] Ahn J-E, Chen Y, Amrein H. Molecular basis of fatty acid taste in drosophila. Elife. 2017;6:e30115. 29231818 10.7554/eLife.30115 PMC5747521

[113] Pellegrino M, Steinbach N, Stensmyr MC, Hansson BS, Vosshall LB. A natural polymorphism alters odour and DEET sensitivity in an insect odorant receptor. Nature. 2011;478:511–4. 21937991 10.1038/nature10438 PMC3203342

[114] Syed Z, Pelletier J, Flounders E, Chitolina RF, Leal WS. Generic insect repellent detector from the fruit fly Drosophila melanogaster. PLoS ONE. 2011;6:e17705. 21436880 10.1371/journal.pone.0017705 PMC3059203

[115] Stensmyr MC, et al. A conserved dedicated olfactory circuit for detecting harmful microbes in Drosophila. Cell. 2012;151:1345–57. 23217715 10.1016/j.cell.2012.09.046

[116] Wang Q, et al. Identification of multiple odorant receptors essential for pyrethrum repellency in Drosophila melanogaster. PLoS Genet. 2021;17:e1009677. 34237075 10.1371/journal.pgen.1009677 PMC8291717

[117] Ai M, et al. Acid sensing by the Drosophila olfactory system. Nature. 2010;468:691–5. 21085119 10.1038/nature09537 PMC3105465

[118] Ai M, Blais S, Park J-Y, Min S, Neubert TA, Suh GS. Ionotropic glutamate receptors IR64a and IR8a form a functional odorant receptor complex in vivo in Drosophila. J Neurosci. 2013;33:10741–9. 23804096 10.1523/JNEUROSCI.5419-12.2013 PMC3693055

[119] Abuin L, Bargeton B, Ulbrich MH, Isacoff EY, Kellenberger S, Benton R. Functional architecture of olfactory ionotropic glutamate receptors. Neuron. 2011;69:44–60. 21220098 10.1016/j.neuron.2010.11.042 PMC3050028

[120] Vulpe A, Menuz K. Ir76b is a co-receptor for amine responses in Drosophila olfactory neurons. Front Cell Neurosci. 2021;15:759238. 34867202 10.3389/fncel.2021.759238 PMC8635857

[121] Prieto-Godino LL, et al. Olfactory receptor pseudo-pseudogenes. Nature. 2016;539:93–7. 27776356 10.1038/nature19824 PMC5164928

[122] Dweck HK, Ebrahim SA, Farhan A, Hansson BS, Stensmyr MC. Olfactory proxy detection of dietary antioxidants in Drosophila. Curr Biol. 2015;25:455–66. 25619769 10.1016/j.cub.2014.11.062

[123] Oh SM, Jeong K, Seo JT, Moon SJ. Multisensory interactions regulate feeding behavior in Drosophila. Proc Natl Acad Sci. 2021;118:e2004523118. 33558226 10.1073/pnas.2004523118 PMC7896327

[124] Jones WD, Cayirlioglu P, Grunwald Kadow I, Vosshall LB. Two chemosensory receptors together mediate carbon dioxide detection in Drosophila. Nature. 2007;445:86–90. 17167414 10.1038/nature05466

[125] Kwon JY, Dahanukar A, Weiss LA, Carlson JR. The molecular basis of CO2 reception in Drosophila. Proc Natl Acad Sci. 2007;104:3574–8. 17360684 10.1073/pnas.0700079104 PMC1805529

[126] Suh GS, et al. A single population of olfactory sensory neurons mediates an innate avoidance behaviour in Drosophila. Nature. 2004;431:854–9. 15372051 10.1038/nature02980

[127] Grosjean Y, et al. An olfactory receptor for food-derived odours promotes male courtship in Drosophila. Nature. 2011;478:236–40. 21964331 10.1038/nature10428

[128] Lin C-C, Prokop-Prigge KA, Preti G, Potter CJ. Food odors trigger Drosophila males to deposit a pheromone that guides aggregation and female oviposition decisions. Elife. 2015;4:e08688. 26422512 10.7554/eLife.08688 PMC4621432

[129] Min S, Ai M, Shin SA, Suh GS. Dedicated olfactory neurons mediating attraction behavior to ammonia and amines in Drosophila. Proc Natl Acad Sci. 2013;110:E1321–9. 23509267 10.1073/pnas.1215680110 PMC3619346

[130] van Naters WvdG, Carlson JR. Receptors and neurons for fly odors in Drosophila. Curr Biol. 2007;17:606–12. 17363256 10.1016/j.cub.2007.02.043 PMC1876700

[131] Auer TO, et al. Olfactory receptor and circuit evolution promote host specialization. Nature. 2020;579:402–8. 32132713 10.1038/s41586-020-2073-7 PMC7100913

[132] Chang H, et al. A chemical defense deters cannibalism in migratory locusts. Science. 2023;380:537–43. 37141362 10.1126/science.ade6155

[133] Matsunaga T, et al. Evolution of olfactory receptors tuned to mustard oils in herbivorous Drosophilidae. Mol Biol Evol. 2022;39:msab362. 34963012 10.1093/molbev/msab362 PMC8826531

[134] Guo H, et al. Sex pheromone communication in an insect parasitoid, Campoletis chlorideae Uchida. Proc Natl Acad Sci. 2022;119:e2215442119. 36442117 10.1073/pnas.2215442119 PMC9894188

